# Spatial and temporal VEGF receptor intracellular trafficking in microvascular and macrovascular endothelial cells

**DOI:** 10.1038/s41598-021-96964-7

**Published:** 2021-08-30

**Authors:** Juliete A. F. Silva, Xiaoping Qi, Maria B. Grant, Michael E. Boulton

**Affiliations:** Department of Ophthalmology and Visual Sciences, University of Alabama at Birmingham, 1670 University Blvd, Volker Hall, Room 472, Birmingham, AL 35233 USA

**Keywords:** Growth factor signalling, Angiogenesis, Cell signalling, Cellular imaging

## Abstract

The vascular endothelial growth factor receptors (VEGFRs) can shape the neovascular phenotype of vascular endothelial cells when translocated to the nucleus, however the spatial and temporal changes in the intracellular distribution and translocation of VEGFRs to the nucleus and the organelles involved in this process is unclear. This study reports the effect of exogenous VEGF on translocation of VEGFRs and organelles in micro- and macrovascular endothelial cells. We showed that VEGF is responsible for: a rapid and substantial nuclear translocation of VEGFRs; VEGFR1 and VEGFR2 exhibit distinct spatial, temporal and structural translocation characteristics both in vitro and in vivo and this determines the nuclear VEGFR1:VEGFR2 ratio which differs between microvascular and macrovascular cells; VEGFR2 nuclear translocation is associated with the endosomal pathway transporting the receptor from Golgi in microvascular endothelial cells; and an increase in the volume of intracellular organelles. In conclusion, the nuclear translocation of VEGFRs is both receptor and vessel (macro versus micro) dependent and the endosomal pathway plays a key role in the translocation of VEGFRs to the nucleus and the subsequent export to the lysosomal system. Modulating VEGF-mediated VEGFR1 and VEGFR2 intracellular transmigration pathways may offer an alternative for the development of new anti-angiogenic therapies.

## Introduction

The VEGF family includes VEGF-A, -B, -C, -D, -E and placental growth factor (PIGF) which exist in a number of different isoforms with varying potency and specificity dependent on local environmental cues and the particular vascular cell type involved. The VEGF receptors are part of the tyrosine kinase receptor (RTK) family; VEGFR1 (Flt-1), VEGFR2 (denoted KDR in human and Flk1 in mouse), and VEGFR3, show different specificity for VEGF family members. VEGF-A (hereafter referred to as VEGF) binds to VEGFR1 with higher affinity than VEGFR2^1–3^. The classic view is that VEGFR2 regulates endothelial function and survival via a number of different canonical signaling pathways^3^ and its signaling and activation is linked to tumor progression^4–6^ and pathological neovascularization^7–9^. Thus blocking this VEGF-mediated signaling pathway is the focus of current anti-angiogenic therapies^2,10–15^. By contrast, less is known about the angiogenic role of VEGFR1 which has weaker kinase activity that VEGFR2^1^. VEGFR1 can be both a positive and negative regulator of angiogenesis dependent on the particular ligand binding^16,17^. Furthermore, VEGFR1 can heterodimerize with VEGFR2^1,18^ and can act as a negative regulator of VEGFR2^1,2^.

There is a growing body of evidence that the intracellular trafficking of RTKs makes a critical contribution to their cellular function and provides an alternative non-canonical signaling pathway^19–21^. Once internalized, the ligand activated receptor will be routed through several endocytic compartments and, eventually, it will be directed to recycling, translocation to specific intracellular sites or degradation^20,21^. We and others have demonstrated that several RTKs, including VEGFR1 and VEGFR2, translocate to the nucleus, chaperone transcription factors or other proteins such as eNOS or caveolin-1 or serve directly as transcription regulators^9,16,19,22–24^. However, the mechanism by which VEGFR1 and VEGFR2 undergo nuclear translocation and the functional activity/role of this process in the nucleus is not fully understood.

The aim of this study was to compare the spatial and temporal changes in the cellular distribution and translocation of VEGFRs in micro- and macrovascular endothelial cells following VEGF treatment.

## Material and methods

### Cell culture

Primary human retinal microvascular endothelial cells (HREC) (Cell Systems Company, Seattle, WA, USA) from 3 different donors and primary normal human aortic endothelial cells (HAEC) (ATCC, Manassas, VA, USA) from 2 different donors were obtained commercially at passage 3 and were cultivated with microvascular endothelial cell basal medium (VBM) [MCDB131 without glutamine (Thermo Fisher Scientific, Grand Island, NY, USA)] supplemented with Microvascular Growth Supplement [MVGS (Thermo Fisher Scientific)] at 37 °C in a 95% air/5% CO_2_ incubator. Cells were used between passages 5–8 for all experiments.

### Cell treatments

HREC and HAEC were cultured in 24 well plates on coverslips precoated with endothelial attachment factor solution (Cell Systems Company). For VEGF treatment, near confluent endothelial cell cultures were washed 2 times in vascular basal media (VBM) and maintained without growth factors in serum-free medium for 45 min. Recombinant human VEGF165 (R&D Systems, Minneapolis, MN, USA) was added at 100 ng/ml to VBM while controls (NO-VEGF) received vehicle alone (VBM) and cells were incubated for 10, 30 or 120 min. For biosynthetic pool depletion experiments, near confluent endothelial cell cultures were washed 2 times in vascular basal media (VBM) and maintained without growth factors in serum-free medium containing 1ug/ml Brefeldin A (Tocris, Bristol, United Kingdom) or Dimethyl sulfoxide vehicle (DMSO- Corning Life Sciences, Union City, CA, USA) diluted 1:7000 during 60 min. Recombinant human VEGF165 (R&D Systems, Minneapolis, MN, USA) was added at 100 ng/ml to VBM containing Brefeldin A or vehicle-DMSO while controls (NO-VEGF) do not received VEGF and cells were incubated for 10, 30 or 120 min. All treatments were made in triplicate for each time point and each experiment was repeated at least 3 times using 3 different cell passage for each donor.

We used VEGFA at 100 ng/ml as this was consistent with our previous studies^16,22,25^. We have looked at lower concentrations and 10 ng/ml gives a similar response to 100 ng/ml VEGFA (Supplementary Fig. 1).

### Immunofluorescence

Cells were fixed with 2% paraformaldehyde in phosphate-buffered saline (PBS) pH 7.4 for 10 min at 37 °C, 2 times washed with PBS and permeabilized with 0.5% triton X-100 (Sigma-Aldrich, Saint Louis, MO, USA) for 5 min. Nonspecific binding sites were blocked for 1 h with 3% bovine serum albumin [(BSA) Sigma-Aldrich] diluted in PBS. The cells were then incubated overnight at 4 °C with primary antibodies: rabbit anti-VEGFR1 C-terminus (#ab2350 Abcam Inc., Cambridge, MA, USA) diluted 1:100, rabbit anti-VEGFR1 N-terminus (#ab32152 Abcam) diluted 1:100, mouse anti-VEGFR1 full length (#H00002321-B01P Abnova, Taipei City, Taiwan) diluted 1:100, rabbit anti-VEGFR2 C-terminus (#2479 Cell Signaling Technology, Danvers, MA, USA) diluted 1:100, rabbit anti-VEGFR2 N-terminus (#ab45010 Abcam) diluted 1:100, mouse anti-CD107a-Lamp-1 (#14107980 eBioscience, San Diego, CA, USA) diluted 1:100, mouse anti-p230 trans Golgi (#611280 BD Biosciences Pharmigen, San Diego, CA, USA) diluted 1:100, sheep anti-calreticulin (#PA131288 Thermo Fisher Scientific) diluted 1:100, sheep anti-EEA1 (#AF8047 R&D Systems) diluted 1:100 in PBS containing 1% BSA and 0.1% Tween 20. After repeated washings with PBS containing 0.1% Tween 20, cells were incubated with the following secondary antibodies: Alexa Fluor 488-conjugated donkey anti-rabbit Ig (Thermo Fisher Scientific) diluted 1:500, Alexa Fluor 555-conjugated donkey anti-mouse Ig (Thermo Fisher Scientific) diluted 1:500, Alexa Fluor 647-conjugated donkey anti-sheep Ig (Thermo Fisher Scientific) diluted 1:500. Hoechst 33342 (Thermo Fisher Scientific) was used to counterstain the cell nuclei. The coverslips were mounted on slides using prolong diamond antifade mounting solution (Thermo Fisher Scientific). Negative controls consisted of reactions from which the primary antibody incubation step was omitted.

### Median intensity of fluorescence and colocalization analysis

Following immunostaining, a minimum of 3 images per coverslip (n = 3 coverslips for each time point and the experiment was repeated at least 3 times per donor) were captured with an A1R-HD25 Ti2 eclipse inverted confocal microscope (Nikon Group Companies, Nanjing, China) using 40 × objective for median intensity fluorescence (MIF) analysis. The images were performed in z-stack, using the same laser excitation and gain settings for each experiment. NIS-Elements software (version 5.0) (https://www.microscope.healthcare.nikon.com) (Nikon Systems Inc., Tokyo, Japan) was used to measure each image: MIF of VEGFRs and organelles, cytoplasmic and nuclear areas stained by VEGFRs, total areas stained by VEGFRs and organelles, number of each stained organelle and total number of nuclei used to relativize the measurements/number of cells/ picture. The fluorescence volume was calculated by multiplying the MIF of each cell region by its respective area and after that divided by the total number of cells in the image. For each experiment all channels fluorescence volume was normalized by the control samples that were not treated with VEGF. The colocalization analysis of the VEGFR1 or VEGFR2 with lysosomes, early endosomes, trans Golgi and Endoplasmic Reticulum (ER) was performed in three ways: 1. Quantifying co-occurring voxels between organelles and receptors in the region of interest (ROI) by Pearson's correlation coefficient; 2. Creating a mask that allows the visualization of co-occurring voxels in ROI determined by quantification in 1; and 3. By quantifying the number of voxels set in 2, which was normalized by the cell volume of each image. The colocalization analysis was made with Imaris Software (version 9.5) (https://imaris.oxinst.com) (Oxford Instruments, Belfast, UK) using the whole 3D volume of each image. At least 3 images for each experiment were made in Z-stack using a 60 × objective for single cell 3D rendering representative images.

### Enzyme-linked immunosorbent assay (ELISA)

For protein extraction, cells from 150 cm^2^ flasks were treated with 0.25% trypsin for 2 min, removed carefully with a cell scraper, washed with PBS and fractions of nuclear and cytoplasmic proteins were obtained using the NE-PER nuclear and cytoplasmic extractions reagents (Thermo Fisher Scientific) according to the manufacturer’s instructions. Total protein was quantified with the BCA protein assay (Thermo Fisher Scientific), using bovine serum albumin as a standard.

The sandwich ELISAs use full-length capture antibody VEGFR2 and N-terminal VEGFR1 and detection C-terminal antibody for both receptors. Total VEGFR levels were quantified using Quantikine sandwich ELISA for Human VEGFR1 (#DVR100C R&D) and PathScan sandwich ELISA for Human VEGFR2 (#7340 Cell signaling) and phosphorylated VEGFRs were quantified using DuoSet IC sandwich ELISA for Human Phospho-VEGFR1 (#DYC4170 R&D) and PathScan sandwich ELISA for Human Phospho-Y1175-VEGFR2 (#7335 Cell signaling) according to the manufacturer’s instructions. Briefly, 10 µg (total VEGFRs quantification) and 25 ug (phospho-VEGFRs quantification) of nuclear and cytoplasmic fraction protein per sample were plated in 96-well flat-bottom plates coated with capture antibodies and nonspecific binding sites blocked, incubated overnight at 4 °C, washed, incubated with peroxidase-conjugated detection antibody and subsequently with substrate solution and absorbance read in a spectrophotometer at 450 nm. The experiments were carried out in triplicate and were repeated 3 times.

The amount of total VEGFRs and phospho-VEGFRs were expressed by optical density (OD) and phospho-VEGFRs were also determined relative to total protein content in the nuclear or cytoplasmic fractions.

### Animals

Forty-eight, 7 weeks old, female C57BL/6 J mice were purchased from Jackson Laboratories (Bar Harbor, ME). All animal procedures were performed in accordance with the National Institutes of Health Guide for Care and Use of Laboratory Animals and the ARVO Statement for the Use Animals in Ophthalmic and Vision Research. All studies were conducted in accordance with Animal Research: Reporting of Vivo Experiments (ARRIVE) guidelines and approved protocols by the Institutional Animal Care and Use Committees from University of Alabama at Birmingham (IACUC protocol # 20826).

### Intravitreal injection

Mice were anesthetized by intraperitoneal injection of ketamine (72 mg/kg)/xylazine (4 mg/kg) and local eye anesthetic with proparacaine HCl was applied topically to the cornea. Animals received intravitreal injection of the recombinant murine VEGF-A_165_ (Thermo Fisher Scientific) 10 ng diluted in 1 µl of 0.9% saline in the left eye and 0.9% saline vehicle control (1 µl) in the right eye. Mice were sacrificed at 2, 6 and 24 h after injection by isoflurane followed by cervical dislocation. N = 3 animals per time point/group.

### Transmission electron microscopy (TEM)

After euthanasia, mouse eyes were enucleated and immediately fixed in 4% paraformaldehyde diluted in PBS pH 7.4 for 30 min followed by immersion fixation in 0.25% glutaraldehyde in 0.1 M sodium cacodylate-HCl buffer pH 7.4 for 2 h at room temperature. Samples were dehydrated through an ethanol series, infiltrated in LR-white (#18181, Ted Pella, Redding, PA), and embedded in LR-white resin that was polymerized at 50 °C overnight. For immunocytochemistry, ultrathin Sects. (90 nm) were placed on nickel grids. Nonspecific binding of antibodies was blocked by 5% normal goat serum and 2% nonfat dried milk diluted in 0.01 M Tris-buffered saline (TBS) pH 7.2. Samples were then incubated for 4 h at room temperature with rabbit anti-VEGFR1 C-terminus (#ab2350 Abcam) or a mouse anti-VEGFR2 N-terminus (# ab42228 Abcam). After 5 washes in PBS, the specimens were incubated with the secondary goat anti-rabbit or anti-mouse IgG antibodies conjugated to 6 or 10 nm gold [#25123 EM-grade 6 nm GAM, #25108 EM-grade 10 nm GAR (Electron Microscopy Sciences, Hatfield, PA, US)]. After washes in buffer, grids were rinsed in de-ionized water and immunolabeled specimens were photographed with or without out post staining using a Tecnai Spirit T12 Transmission Electron Microscope (Thermo-Fisher, formerly FEI) operating at 80 kV equipped with a high speed 29 Megapixel TEM camera (Advanced Microscopy Techniques, Corp). The endothelial cells were viewed in the entire retina fitted on the EM grid with 300 mesh holes. Photographs were taken at a magnification of 6,500X to 11,000X. Immunogold particles in the cytoplasm or nuclei of retinal microvascular endothelial cells were manually quantitated from at least 10 endothelial cells for each experiment and correlated with the respective area in which it was found. Mean values are expressed as the number of gold particles per 5 µm^2^ after analysis using a NIH ImageJ software (version 1.52i) (http://imagej.nih.gov/ij). The immunoglod experiment was repeated 2 times for each animal (n = 3 mice /time point group). Thus a total of 60 cells were analyzed per time point/group.

### Statistical analysis

In vitro experiments were repeated at least three times per cell donor and in vivo experiment was repeated twice. Statistical analyses were performed by 1-way ANOVA and multi-comparison post-hoc Tukey’s test was used to determine intra-group differences with respect to the time of treatment for all median intensity of immunofluorescence (MIF), colocalization analysis, immunogold transmission electron microscopy and ELISAs. 2-way ANOVA followed by multi-comparison post-hoc Tukey’s and t-student tests were used to determine intra-group differences with respect to the time and type of treatment with Vehicle and Brefeldin A. GraphPad Prism software (version 8.1.2) (https://www.graphpad.com) was used for the calculations and visualization. Results are expressed as mean ± SEM and *p* < 0.05 was considered as statistically significant.

## Results

**Figure 1:**
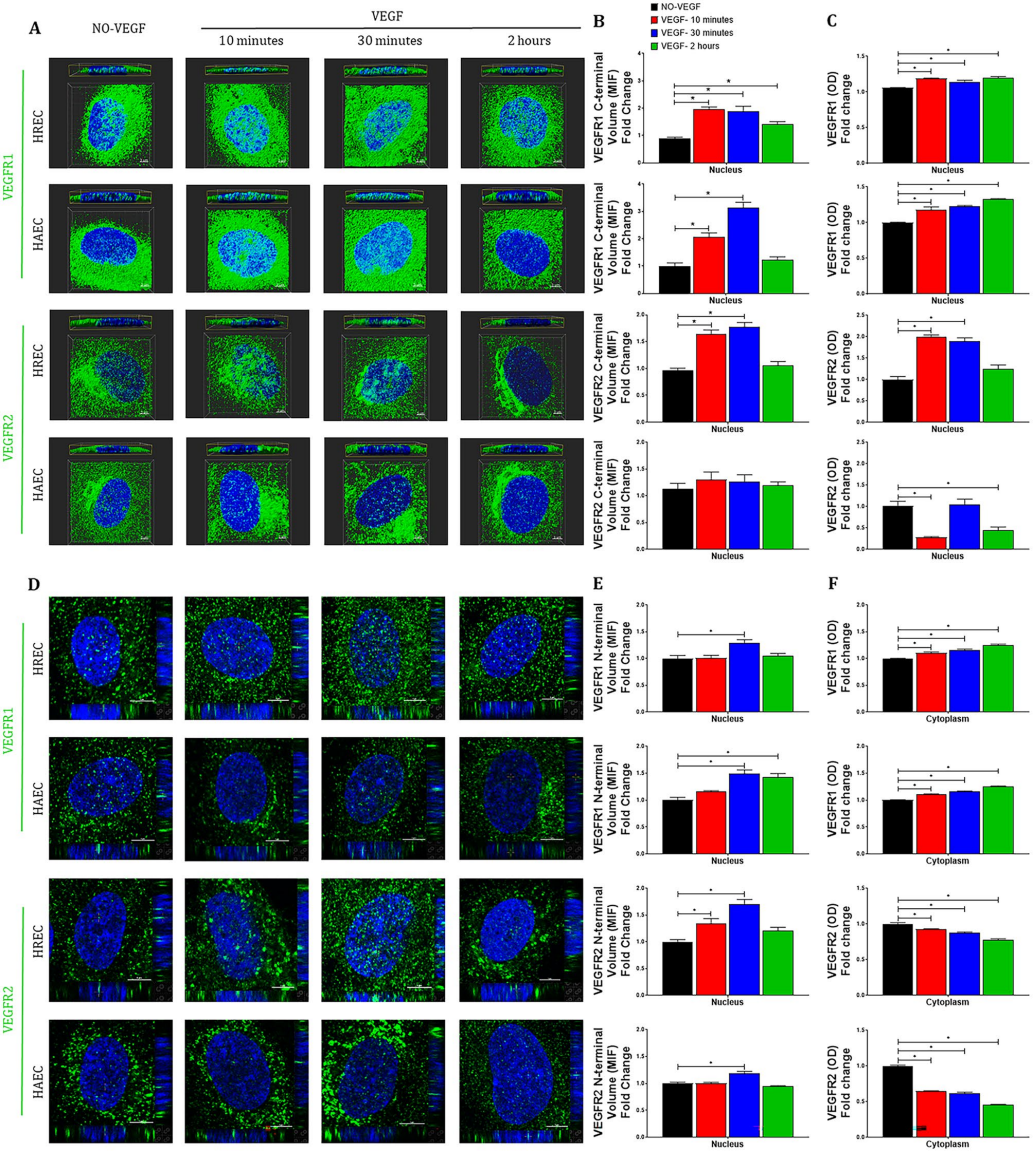
**VEGF-induced VEGFR migration to the nucleus in micro- and macrovascular endothelial cells**. The nuclear and cytoplasmic levels of VEGFR1 and VEGFR2 in HREC and HAEC were evaluated by immunofluorescence plus sandwich ELISA at different time points (10 min, 30 min and 2 h) after exposure to 100 ng/ml VEGF and compared to saline controls (NO-VEGF). Representative 3D reconstruction rendering images of the HREC and HAEC after VEGF treatment and control immunostained for (**A**) C-terminal VEGFR1 and VEGFR2. The top image of each block is the horizontal section of the 3D rendering below used as a way of showing the amount of the respective receptor inside the nucleus. Scale bars = 3 μm, and (**D**) N-terminal VEGFR1 and VEGFR2. The top left image of each block shows the position of the VEGFR1 nuclear translocation in only one focal plane and the correlation of this point are located in the 3D lateral and lower cross sections. Scale bars = 5 μm. (**B** and **E**) Graphs showing the quantification of the median intensity of fluorescence (MIF) volume of at least 10 confocal z-stack images at 400 × magnification for each experiment performed using NIS elements software. (**C** and **F**) Graphs showing the quantification of the total VEGFR1 and VEGFR2 present in nuclear and cytoplasmic fractions of the HREC and HAEC after VEGF treatment and control quantified by sandwich ELISA. All graphs represent VEGF time point fold change with respect to control NO-VEGF and the graphs bars represents the average of the a minimum of 3 separate experiments from 3 donors of the HREC and 2 donors of the HAEC. * = *p* < 0.05 as determined by 1-way ANOVA and multi-comparison post-hoc Tukey’s test was considered significant.

To evaluate the time-dependent nuclear translocation of VEGFR1 and VEGFR2 following VEGF stimulation we determined the cytoplasmic and nuclear levels of VEGFR1 and VEGFR2 after VEGF treatment by quantitative immunofluorescence and sandwich ELISA (Fig. 1).

### VEGF stimulates a rapid and significant nuclear translocation of VEGFR1 in both microvascular and macrovascular endothelial cells

The VEGFR1 (C-terminal and N-terminal portions) staining showed that VEGFR1 was at relatively low levels in the nucleus of control untreated HREC and HAEC with lower levels of VEGFR1 in the plasma membrane and higher levels in the cytoplasm (Fig. 1A and F). By contrast, following 10 and 30 min of VEGF treatment a significant translocation of VEGFR1 to the nucleus (Fig. 1A–E) in both HREC and HAEC which was associated with decreased VEGFR1 staining in the plasma membrane (Fig. 1A and 1D) but not VEGFR1 in cytoplasmic fractions (Fig. 1A, D and F). Nuclear C-terminal VEGFR1 density had a maximal increase of twofold in HREC and more than threefold in HAEC compared to untreated control between 10 and 30 min following VEGF treatment (Fig. 1B), as well as the nuclear protein fractionation showed a 20% increase in the amount of total VEGFR1 (Fig. 1C) in the same period of time in both, HREC and HREC similar to nuclear N-terminal VEGFR1 density in HREC that had transient 20% increase only at 30 min while HAEC achieved a maximum 50% increase in N-Terminal VEGFR1 levels in the nucleus at 30 min after VEGF treatment (Fig. 1E).

Interestingly, for the HREC there was some donor to donor variation in nuclear translocation following VEGF treatment with respect to both the timing and level of C-terminal VEGFR1 in the nucleus: two donors peaked at 10 min and the other at 30 min with this latter donor showing the highest levels of VEGFR1 in the nucleus (Supplementary Fig. 2A).

At 2 h following VEGF treatment in HREC, nuclear C-terminal VEGFR1 density was decreased by approximately 50% compared to that at 10- and 30-min post treatment (Fig. 1A–B), nuclear VEGFR1 protein levels remained increase 20% compared to the VEGF-untreated control (Fig. 1C) and N-terminal VEGFR1 density returned to basal levels (Fig. 1E–F). By contrast, in HAEC nuclear C-terminal VEGFR1 rapidly decreased to near basal levels in untreated controls (Fig. 1A–B), nuclear protein levels as well as N-terminal VEGFR1 remained increased 30% and 45% compared to the VEGF-untreated control, respectively (Fig. 1C and F). Overall, nuclear translocation of the C-terminal VEGFR1 was 80% greater than for the N-terminal suggesting that the majority of the nuclear VEGFR1 is in a truncated form without the N-terminus.

### VEGF stimulates rapid nuclear translocation of VEGFR2 in microvascular, but not macrovascular endothelial cells

C-terminal and N-terminal portions of the VEGFR2 in control untreated HREC and HAEC was localized to the cytoplasm and plasma membrane but was at very low levels in the nucleus (Fig. 1A and D). Following VEGF treatment, a significant translocation of VEGFR2 to the nucleus was observed (Fig. 1A and D) and quantified (Fig. 1B–C and E) in HREC, but not in HAEC. Nuclear translocation of both C-terminal, total and N-terminal VEGFR2 in HREC was maximal between 10 and 30 min and similar (twofold) following VEGF treatment (Fig. 1B–C and E), which was associated with decreased VEGFR2 levels in the cytoplasm (Fig. 1F). By contrast, there was no significant nuclear translocation of C-terminal and total VEGFR2 in HAEC following VEGF treatment at any of the time points examined and levels remained similar or lesser to untreated control cells (Fig. 1B–C) and only a significant 17% increase in the N-terminal VEGFR2 density after 30 min of VEGF treatment (Fig. 1E).

However, the increased nuclear levels of C-terminal, total and N-terminal VEGFR2 in HREC and N-terminal VEGFR2 in HAEC were transient and returned to baseline after 2 h following VEGF treatment similar to that in unstimulated controls (Fig. 1B–C and E), but the cytoplasmic pool of VEGFR2 remained decreased at this time point in both micro- and macro vascular endothelial cells compared to control NO-VEGF untreated (Fig. 1F). Both the timing and level of C-terminal VEGFR2 in the nucleus following VEGF treatment was similar between donors (Supplementary Fig. 2B).

### Nuclear translocation of VEGFRs is observed in microvascular endothelial cells in vivo

**Figure 2:**
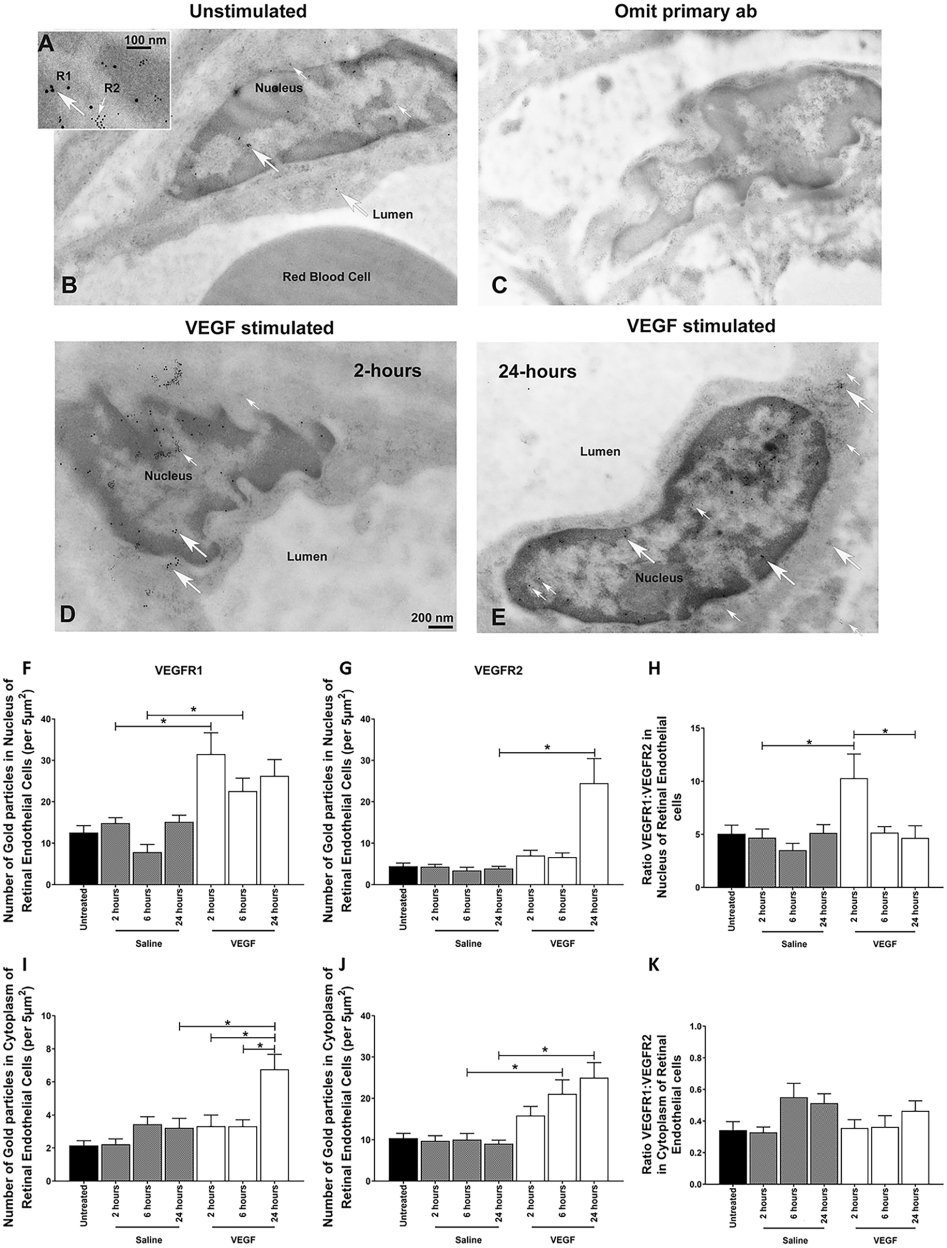
**Intravitreal injection of VEGF promotes nuclear localization of VEGFR1 and VEGFR2 in mouse microavascular retinal endothelial cells**. Electron microscopy (EM) of VEGFR1 and VEGFR2 immunogold staining in C57BL/6 J mouse retinal endothelial cells. The nuclear and cytoplasmic levels of VEGFRs in retinal endothelial cells from C57BL/6 J mice were evaluated at different time points (2, 6 and 24 h – n = 3/time point) after intravitreal injection of 10 ng VEGF. Negative controls were either: a) untreated eyes or b) eyes that received intravitreal injection of the vehicle (1 ul of the 0.9% saline). (**A**) Double immunogold staining was achieved using two different size of the gold particles to differentiate VEGFR1 (10 nm gold) from VEGFR2 (6 nm). (**B**) Representative EM images from retinal endothelial cells from a control unstimulated eye. (**C**) Negative control showing no specific gold particles in a retinal endothelial cell when the primary antibody was omitted. (**D–E**) Representative EM images from retinal endothelial cells from 2 and 24 h VEGF-stimulated eyes showing expression of VEFR1 (large arrows) and VEGFR2 (small arrows). Scale bar = 200 nm. Graphs show the quantification of the number of gold particles per area (5 µm^2^) in the nucleus (**F** and **G**) and cytoplasm (**I** and **J**) of at least 10 endothelial cells at 11,000 × magnification for each experiment performed using ImageJ software. (**H** and **K**) The ratio of VEGFR1:VEGFR2 in the nucleus and cytoplasm. Experiments was repeated 2 times for each animal. * = *p* < 0.05 as determined by 1-way ANOVA and multi-comparison post-hoc Tukey’s test is considered significant.

We next performed an in vivo study to confirm that nuclear translocation was not a phenomenon of cell culture and occurred in vivo. We chose longer time points as this is when we previously see significant retinal vascular permeability following intravitreal injection of VEGF^25^ and a single intravitreal injection of VEGF is insufficient to promote a neovascular response in the retina. Mice received an intravitreal injection of VEGF and the cytoplasmic and nuclear levels of VEGFR1 and VEGFR2 were determined using immunogold staining of retinal endothelial cells vessels imaged by transmission electron microscopy (Fig. 2).

VEGFR1 was localized mainly in the nucleus while VEGFR2 was mainly in cytoplasm of retinal microvascular endothelial cells from unstimulated mice (Fig. 2A–B). There was no significant change in receptor localization following intravitreal saline injection (Fig. 2F–G and I–J). Following intravitreal injection of VEGF into mice a significant 150% and 80% increase in nuclear VEGFR1 was observed in retinal microvascular endothelial cells at 2 and 6 h, respectively compared with eyes that received vehicle alone (Fig. 2F). In contrast, a 450% increase in nuclear VEGFR2 was observed 24 h after VEGF injection into the eye (Fig. 2E and G). These combined data showed that the nuclear VEGFR1:VEGFR2 ratio increased 100% just 2 h following VEGF treatment (Fig. 2D and H). Despite a 100% increase in VEGFR1 (Fig. 2I) and maximal 150% increase in VEGFR2 (Fig. 2J) levels in retinal endothelial cells after VEGF treatment did not result in a significant change in the cytoplasmic VEGFR1:VEGFR2 ratio (Fig. 2K).

### Changes in the VEGFR1:VEGFR2 ratio following VEGF treatment

The temporal and fold-change differences between VEGFR1 and VEGFR2 following VEGF treatment in this and other studies^18,25^ led us to determine how the ratio between the two receptors changes in the nucleus. We thus evaluated if the ratio of VEGFR1:VEGFR2 within the nucleus could be regulated by VEGF using double immunofluorescence staining for both receptors.

**Figure 3:**
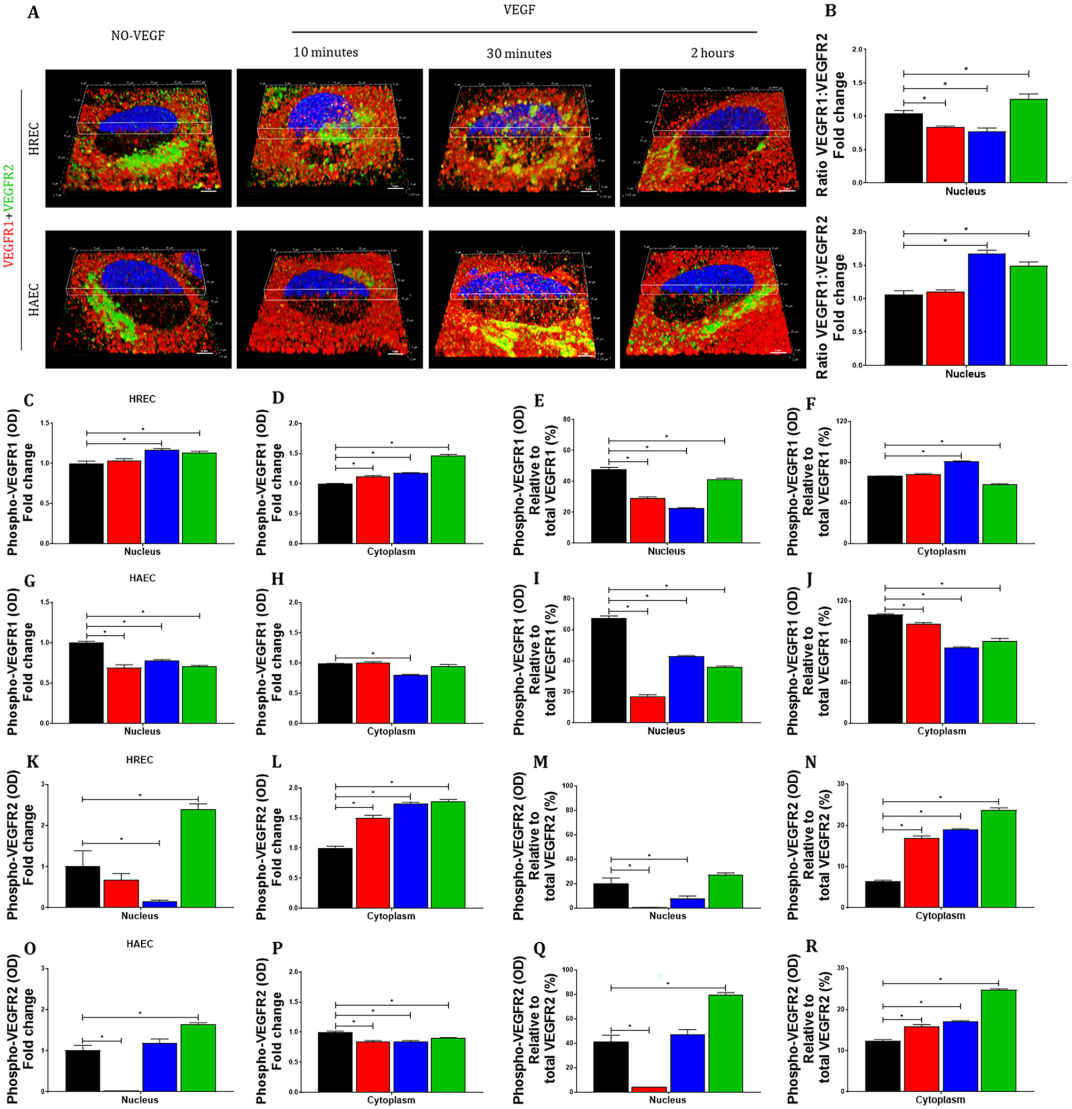
**The nuclear VEGFR1:VEGFR2 ratio is different in micro- and macrovascular endothelial cells and VEGF-stimulated nuclear VEGFRs are minimally phosphorylated**. The nuclear levels of VEGFR1 and VEGFR2 plus of phospho-VEGFR1 and phospho-VEGFR2 in HREC and HAEC were evaluated by double immunofluorescence and sandwich ELISA, respectively, at different time points (10 min, 30 min and 2 h) after exposure to 100 ng/ml VEGF and compared to untreated controls (NO-VEGF). (**A**) Representative 3D reconstruction rendering images of the HREC and HAEC after VEGF treatment and control immunostained for full length VEGFR1 (red) + C-terminal VEGFR2 (green) showing simultaneous nuclear localization of both receptors after VEGF treatment. The nuclei were fluorescently stained with the DNA intercalating dye Hoechst (blue) and the blue channel was partially removed from the 3D representations to evidence the simultaneous localization and association of VEGFR1 and VEGFR2 in the area corresponding to the nucleus. The associated points that spatial colocalize green and red are shown in yellow. Scale bars = 5 μm. (**B**) Graphic representation of the nuclear VEGFR1:VEGFR2 ratio of HREC and HAEC following addition of VEGF shown in A after the quantification of the median intensity of fluorescence (MIF) of at least 10 confocal z-stack images at 400 × magnification for each experiment performed using NIS elements software. VEGFR1:VEGFR2 ratio graphs represent VEGF time point fold change with respect to control NO-VEGF and the graphs bars represents the average of the a minimum of 3 separate experiments from HREC and HAEC. Graphs showing the quantification of the phospho-VEGFR1 (**C–D**, **G–H)** and phospho-VEGFR2 (**K–L**, **O–P**) present in nuclear (**C**, **G**, **K** and **O**) and cytoplasmic (**D, H, L** and **P**) fractions of the HREC and HAEC after VEGF treatment and control quantified by sandwich ELISA. (**E–F**, **I–J**, **M–N**, **Q–R**) graphic representation of the phospho-VEGFR1 and phospho-VEGFR2 percentage relative to total amount of VEGFR1 or VEGFR2 found in the nucleus and cytoplasm of the HREC and HAEC following addition of VEGF. ELISA data for each VEGF time point was normalized by the control NO-VEGF and bars represent the average of the a minimum of 3 separate experiments from 3 donors of the HREC and 2 donors of the HAEC. * = *p* < 0.05 as determined by 1-way ANOVA and multi-comparison post-hoc Tukey’s test was considered significant.

The VEGFR1:VEGFR2 ratio reaches a maximum decrease of ~ 30% between 10 and 30 min of VEGF stimulation in HREC (Fig. 3A–B), unlike HAEC where the nuclear VEGFR1:VEGFR2 ratio does not decrease and culminates in an increase of 70% at 30 min of VEGF treatment (Fig. 3A–B) due to non-migration of VEGFR2 to macrovascular endothelial cells VEGF-dependent (Fig. 1A–C and 3A). In this period of time between 10 and 30 min of stimulation with VEGF, where we had the maximum VEGFR1 and VEGFR2 nuclear migration, although we can easily visualize many points where the 2 receptors are associated (Fig. 3A), the spatial nuclear distribution of VEGFR1 and VEGFR2 is preferably different.

After 2 h of VEGF treatment the VEGFR1:VEGFR2 ratio is increased in both micro- and macrovascular endothelial cells (Fig. 3B) and this was associated with VEGFR2 exit from the HREC nucleus and VEGFR1 nuclear permanence in both cells (Fig. 1A–C and 3A).

### Nuclear VEGFR1 and VEGFR2 are minimally phosphorylated

To analyze if the time-dependent nuclear translocations of VEGFR1 and VEGFR2 following VEGF treatment could be in the phosphorylated form or not we evaluate the phospho-VEGFR1 and phospho-VEGFR2 in the nucleus and this phosphorylated form proportional to the total amount of the same receptors in each time point studied through ELISA.

VEGF treatment increased the nuclear and cytoplasmic levels of phosphorylated VEGFR1 in HREC (Fig. 3C–D), but not in HAEC (Fig. 3G–H). However, when phospho-VEGFR1 levels were normalized relative to the total levels of VEGFR1 found in the nucleus, treatment with VEGF caused a proportional decrease in phospho-VEGFR1 levels in the nucleus of both micro- and macrovascular endothelial cells, showing that although there may be translocation of the phosphorylated form of VEGFR1 to the nucleus, this is minimal.

The nuclear levels of phosphorylated VEGFR2 drastically decreased after 30 and 10 min of VEGF treatment in HREC (Fig. 3K) and HAEC (Fig. 3O), respectively. The proportion of phospho-VEGFR2 relative to the total VEGFR2 in the nucleus (Fig. 3M and Q), is also decreased after 30 and 10 min of VEGF treatment showing that the migration of phospho-VEGFR2 to the nucleus is minimal at most in both micro- and macrovascular endothelial cells.As expected, VEGF stimulation was responsible for a significant and sustained increase in VEGFR2 phosphorylation in the cytoplasm of HREC (Fig. 3N) and HAEC (Fig. 3R) during the entire time period studied.

### VEGF modulates the association between VEGFR1 and intracellular organelles

In an attempt to understand how the cellular machinery would influence the traffic of VEGFRs and perhaps identify a putative mechanistic pattern that would determine a preferential pathway for this nuclear translocation we evaluated: A. temporal and spatial colocalization of VEGFR1 and VEGFR2 with trans Golgi complex, endoplasmic reticulum (ER), early endosomes and lysosomes after VEGF treatment, and B. the influence of VEGF on the cellular machinery through the measurement of organelle volume.

**Figure 4:**
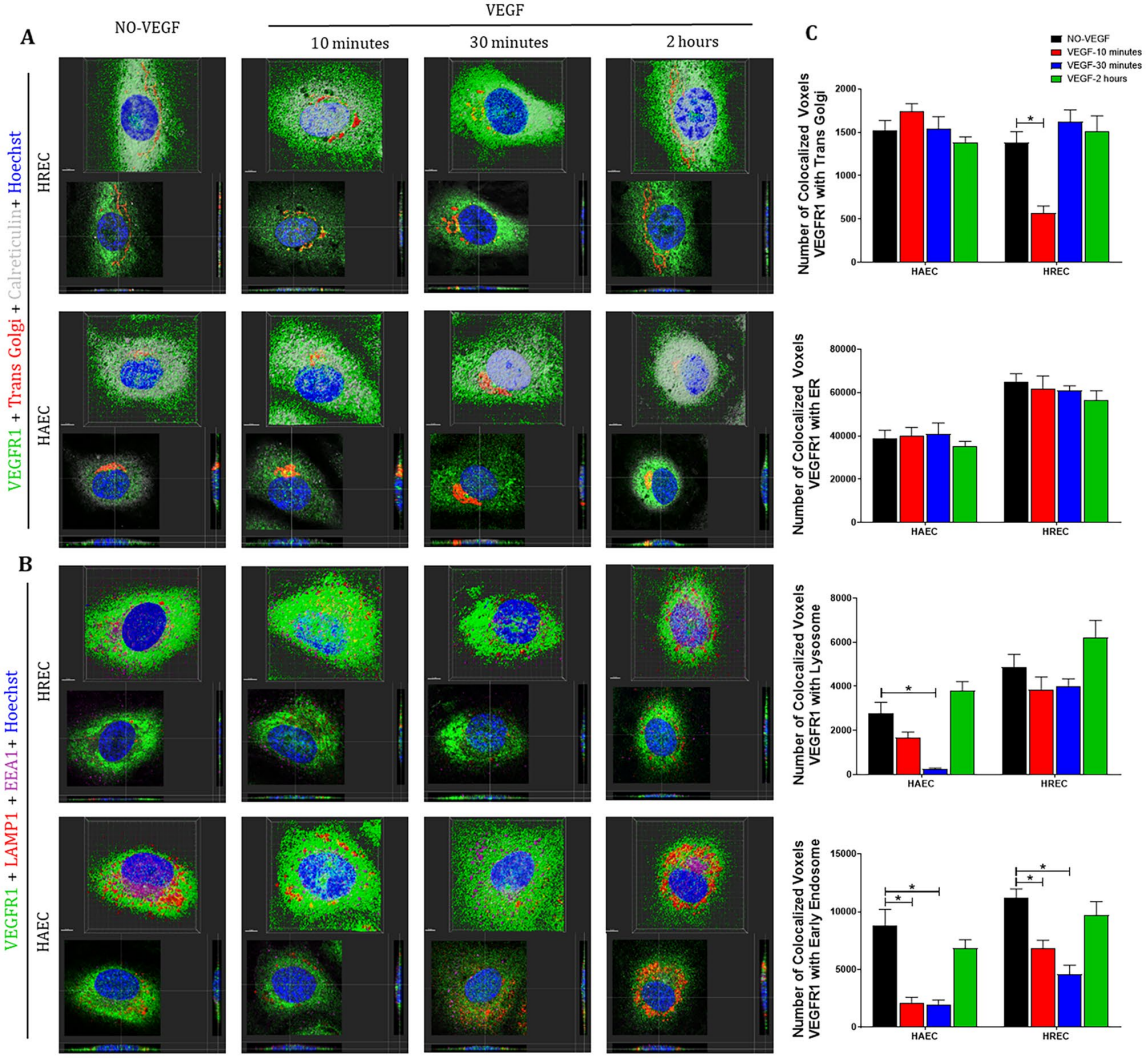
**VEGFR1 translocates from intracellular compartments to the nucleus after VEGF treatment of endothelial cells**. Intracellular trafficking of VEGFR1 in HREC and HAEC were evaluated by immunofluorescence at different time points (10 min, 30 min and 2 h) after exposure to 100 ng/ml VEGF and compared to saline controls (NO-VEGF). Triple immunofluorescence was performed to colocalize VEGFR1 with specific organelles. (**A**) 3D reconstruction rendering of the HREC and HAEC immunostained with VEGFR1 (green), trans Golgi (red) and calreticulin (endoplasmic reticulum-ER gray) (**B**) 3D reconstruction rendering of HREC and HAEC immunostained with VEGFR1 (green), LAMP1 (lysosome-red) and EEA1 (early endosome-magenta). The nuclei in (**A**) and (**B**) were fluorescently stained with the DNA intercalating dye Hoechst (blue). The colocalization points between green and red are shown in yellow and between green and magenta shown in gray. The top image of each block shows the 3D rendering of the representative cells at each time point and the same cells are shown in the lower image of each block showing the position of the nuclear translocation of VEGFR1 in only one focal plane in the center and the correlation of this point located in the 3D lateral and lower cross sections. Scale bars = 5 μm. (**C**) Quantification of the number of VEGFR1 colocalized voxels with the organelles relative to the number of cells in each image. The results show the analyzes of at least 10 confocal z-stack images at 400 × magnification for each time point by Imaris software and summarize the data obtained by quantifying the organelles from a minimum of 3 separate experiments from 3 donors of the HREC and 2 donors of the HAEC. The results show that the colocalization of VEGFR1 with trans Golgi, early endosomes and lysosomes decrease between 10 and 30 min after VEGF treatment, which correlates with the increase of this receptor in the nucleus during the same period. * = *p* < 0.05 as determined by 1-way ANOVA and multi-comparison post-hoc Tukey’s test is considered significant.

VEGFR1 was colocalized to lysosomes, early endosomes, trans Golgi, and ER (Fig. 4A–B) in both control untreated HREC and HAEC. However, after 10 and 30 min VEGF treatment the number of colocalized voxels between VEGFR1 with trans Golgi and early endosomes decreased in HREC while in HAEC the colocalization between VEGFR1 with lysosomes and early endosomes decreased (Fig. 4A–C). The association of the VEGFR1 with the organelles returned to that described in unstimulated controls after 2 h VEGF treatment (Fig. 4A–C).

Although the VEGF treatment had temporally modulated the VEGFR1 number of colocalized voxels with the organelles (Fig. 4C), VEGF did not alter the VEGFR1 spatial localization with organelles in both HREC and HAEC (Fig. 4A–B).

### VEGF differentially modulates the spatial and temporal association of VEGFR2 with organelles in micro- and macrovascular endothelial cells

**Figure 5:**
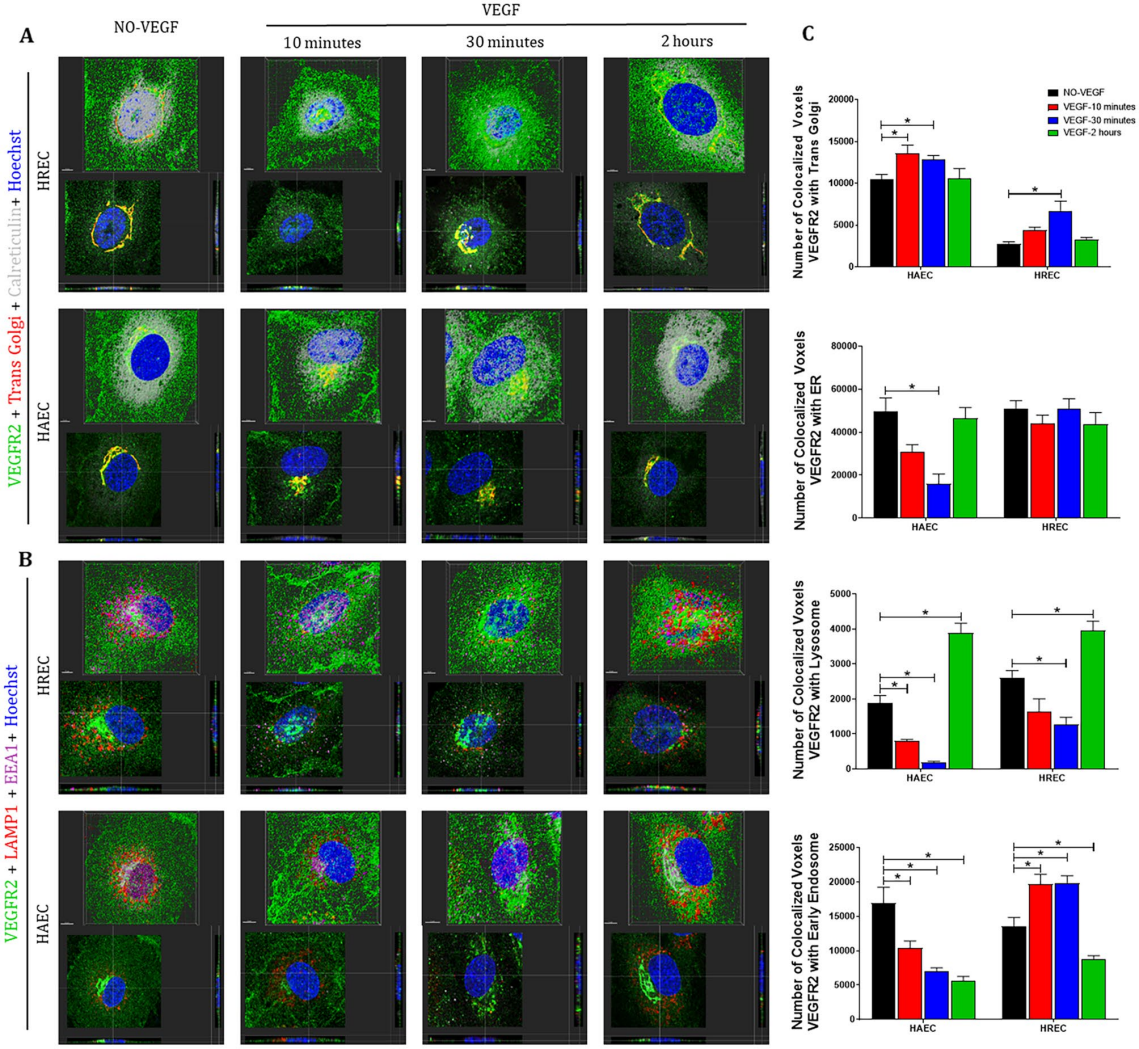
**VEGFR2 can translocate from plasma membrane and intracellular compartments to the nucleus via early endosomes following VEGF treatment in endothelial cells**. Intracellular trafficking of VEGFR2 in HREC and HAEC was evaluated by immunofluorescence at different time points (10 min, 30 min and 2 h) after exposure to 100 ng/ml VEGF and compared to saline controls (NO-VEGF). Triple immunofluorescence was performed to colocalize VEGFR2 with specific organelles. (**A**) 3D reconstruction rendering of the HREC and HAEC immunostained with VEGFR2 (green), trans Golgi (red) and calreticulin (endoplasmic reticulum-ER gray) (**B**) 3D reconstruction rendering of HREC and HAEC immunostained with VEGFR2 (green), LAMP1 (lysosome-red) and EEA1 (early endosome-magenta). The nuclei in (**A**) and (**B**) were fluorescently stained with the DNA intercalating dye Hoechst (blue). The colocalization points between green and red are shown in yellow and between green and magenta shown in gray. The top image of each block shows the 3D rendering of the representative cells of each time point and the same cells are shown in the lower image of each block showing the position of the nuclear translocation of VEGFR2 in only one focal plane in the center and the correlation of this point located in the 3D lateral and lower cross sections. Scale bars = 5 μm. (**C**) Quantification of the number of VEGFR2 colocalized voxels with the organelles relative to the number of cells in each image. The results show the analyzes of at least 10 confocal z-stack images at 400 × magnification for each time point by Imaris software and summarize the data obtained by quantifying the organelles from a minimum of 3 separate experiments from 3 donors of the HREC and 2 donors of the HAEC. The results show that the colocalization of VEGFR2 with trans Golgi and early endosomes increases between 10 and 30 min after VEGF treatment compared to control and correlates with the increase of VEGFR2 in the nucleus in HREC, but not in HAEC. * = *p* < 0.05 as determined by 1-way ANOVA and multi-comparison post-hoc Tukey’s test is considered significant.

Similar to VEGFR1, VEGFR2 was colocalized with lysosomes, early endosomes, trans Golgi, and ER in both control untreated HREC and HAEC (Fig. 5A–B). The colocalization of VEGFR2 with early endosomes was strongly concentrated in the region that corresponds to Golgi apparatus and ER (Fig. 5A). However, unlike what was seen for VEGFR1, the VEGF treatment was responsible for a more rapid concentration, polarization and association of VEGFR2 with early endosomes in the plasma membrane and perinuclear regions, clearly visualized (Fig. 5B) and quantified (Fig. 5C) after 10 and 30 min VEGF treatment in HREC. In contrast, HAEC had a decreased number of colocalized voxels between VEGFR2 and early endosomes after 10–30 VEGF treatment (Fig. 5C). In addition, VEGFR2 and early endosomes were strongly colocalized in the Golgi apparatus region and despite the difference in concentration, the location of this colocalization was not altered by the VEGF treatment (Fig. 5B). At the same time point VEGFR2 showed increased colocalization with Golgi and decreased colocalization with lysosomes in both micro- and macrovascular endothelial cells (Fig. 5C). After 2 h of VEGF treatment, both HREC and HAEC had the perinuclear VEGFR2 colocalization with lysosomes increased (Fig. 5B–C).

### VEGF determines the volume and location of intracellular organelles

**Figure 6:**
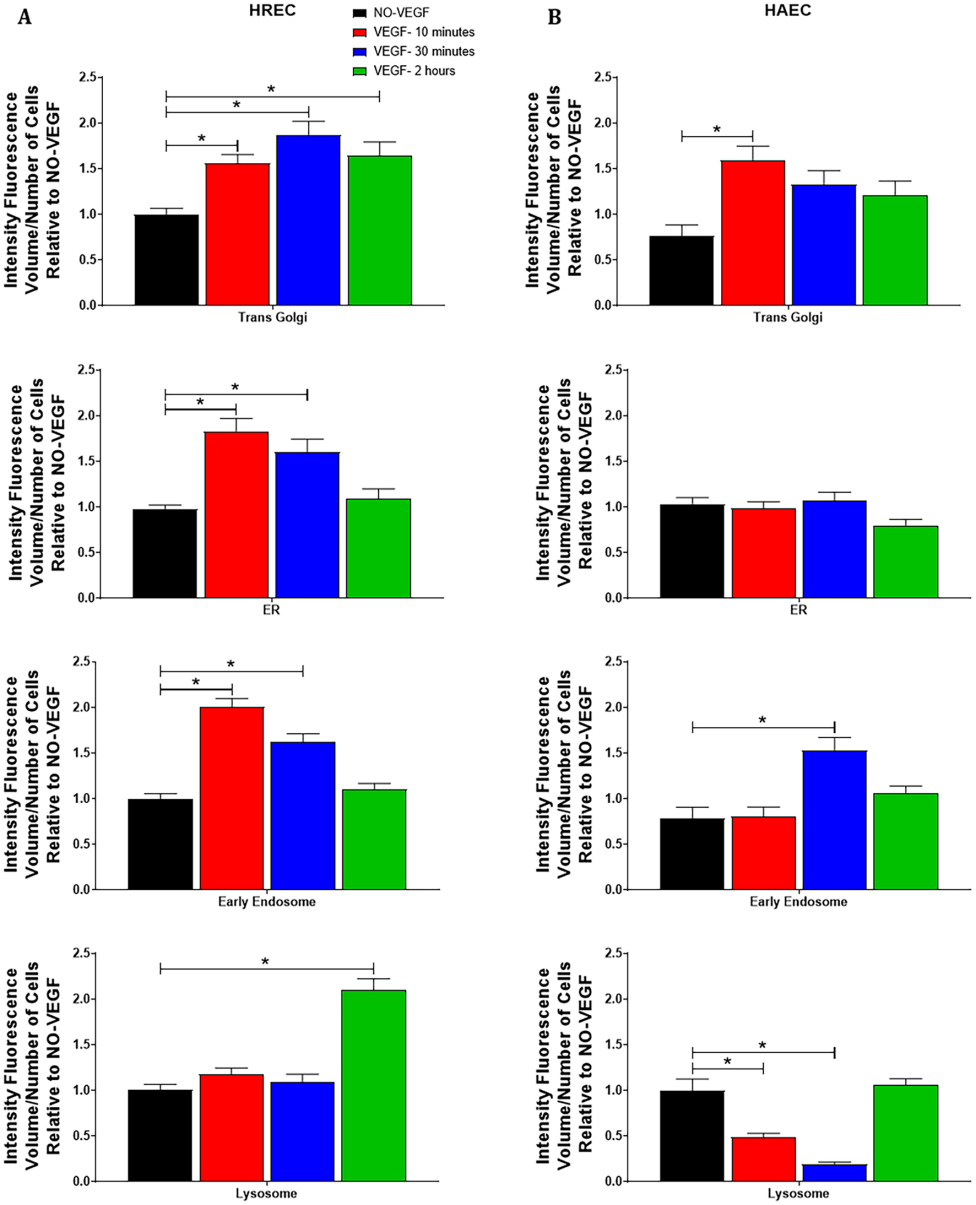
**VEGF changes the volume of intracellular organelles**. Trans Golgi, endoplasmic reticulum (ER), early endosome and lysosome density was measured in HREC and HAEC by the quantification of the MIF relative to the area occupied by each organelle and divided by the number of cells. Graphs depict quantification of organelles density of at least 10 confocal z-stack images at 400 × magnification for each experiment performed using NIS elements software. Data for each VEGF time point was normalized to the control NO-VEGF and the graphs show the average from a minimum of 3 separate experiments from 3 donors of the HREC and 2 donors of the HAEC. * = *p* < 0.05 as determined by 1-way ANOVA and multi-comparison post-hoc Tukey’s test is considered significant.

In untreated controls, early endosomes and lysosomes were extensive and concentrated in the region that corresponds to Golgi apparatus and ER while minimal, dispersed, staining in the cytoplasm was observed for both HREC and HAEC untreated control (Fig. 4A and 5A). VEGF was responsible for rapid spatial changes and polarization of the early endosomes in the plasma membrane and perinuclear regions (Fig. 4A and 5A) and a rapid increase of trans Golgi, ER and early endosomes volumes (Fig. 6A) after 10 and 30 min VEGF treatment in HREC. Interestingly, the same movement and polarization changes of the early endosomes was not seen in HAEC (Fig. 4A and 5A) even though HAEC presented the same increase in trans Golgi and early endosome volumes (Fig. 6B) as seen for HREC. In this same period of time, VEGF was also responsible for spatial changes in the location of the lysosomes that were concentrated in the region that corresponds to the Golgi apparatus and ER before treatment to dispersed in the cytoplasm in both HREC and HAEC between 10 and 30 min of VEGF treatment (Fig. 4A and 5A), whereas in HAEC the volume of lysosomes decreased considerably during this time point (Fig. 6B).

After 2 h of VEGF treatment, early endosomes levels (Fig. 6A–B) and location (Fig. 4A and 5A) were back to those presented in the untreated control for both HREC and HAEC, the trans Golgi volume remains increased in HREC compared to control (Fig. 6A), lysosomes levels (Fig. 6B) and location (Figs. 4A and 5A) were back to those presented in the untreated control in HAEC. However, in HREC the volume of lysosomes was increased 110% compared to control (Fig. 6A) and lysosomes were strongly concentrated at the perinuclear region (Figs. 4A and 5A).

### The VEGF-stimulated VEGFR2 in the nucleus of microvascular endothelial cells comes from Golgi

Due to the strong association of VEGFR2 with Golgi, associated with increased trans Golgi volume, we determined if the VEGFR2 that is going to the nucleus after VEGF stimulation is derived from the Golgi. To test this theory, we treated HREC and HAEC with a reversible inhibitor of protein translocation from ER to Golgi (Brefeldin A) for 1 h before and during VEGF treatment and then we evaluated whether blocking the biosynthetic pool would influence migration of VEGFRs.

**Figure 7:**
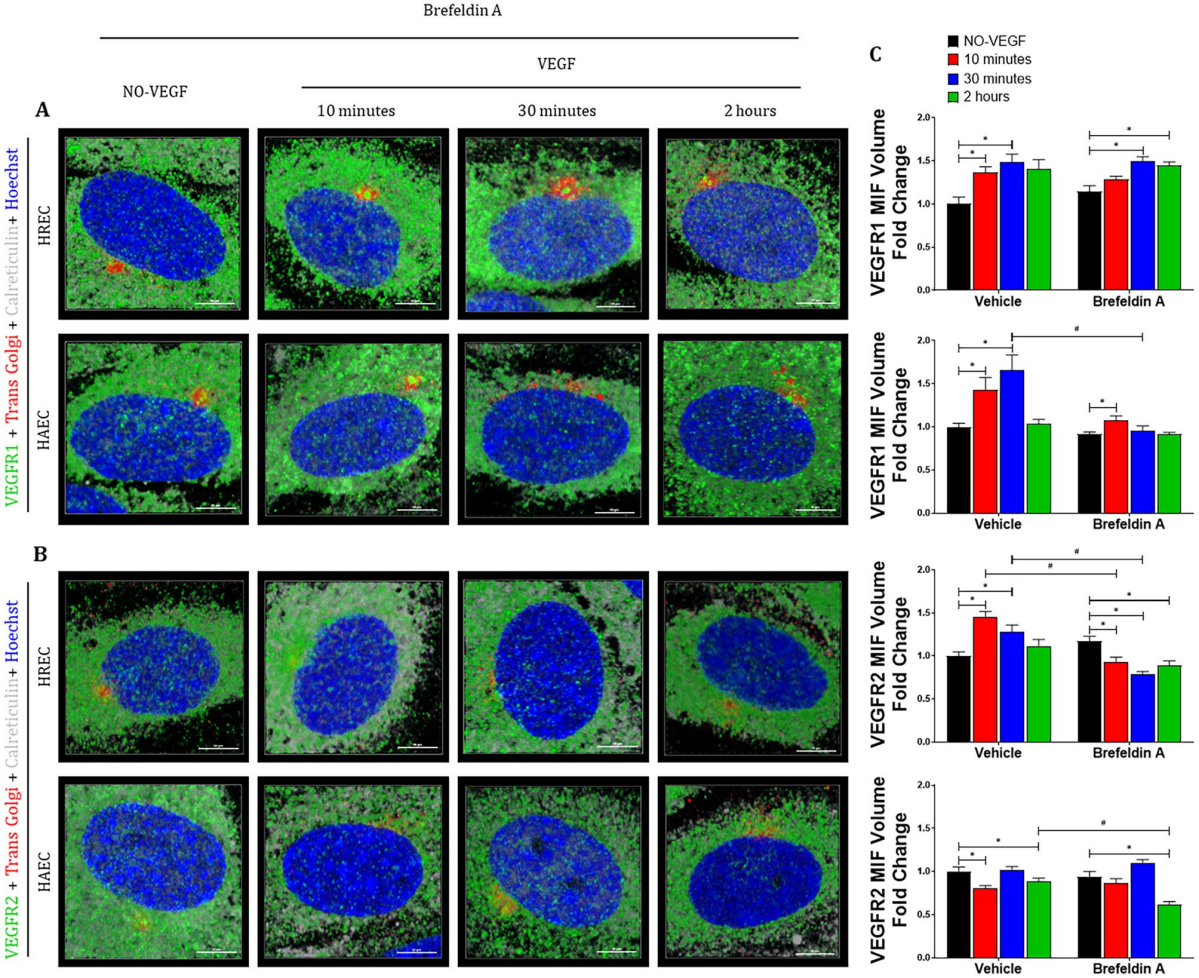
**Depletion of the biosynthetic pool by Brefeldin A prevents nuclear translocation of VEGFR2 into VEGF-stimulated microvascular endothelial cells**. Intracellular trafficking of VEGFR1 and VEGFR2 in HREC and HAEC was evaluated by immunofluorescence at different time points (10 min, 30 min and 2 h) after exposure to 100 ng/ml VEGF and compared to saline controls (NO-VEGF). Triple immunofluorescence was performed to confirm the biosynthetic pool depletion after Brefeldin A treatment and evaluate the nuclear translocation of the VEGFRs by measuring the MIF. (**A–B**) 3D reconstruction rendering of the HREC and HAEC immunostained with VEGFR1 and VEGFR2 (green), respectively, plus trans Golgi (red), calreticulin (endoplasmic reticulum-ER gray) and DNA intercalating dye Hoechst (nuclei-blue). The colocalization points between green and red are shown in yellow and between green and gray shown in cyan. Scale bars = 10 μm. (**C**) Graphs showing the quantification of the median intensity of fluorescence (MIF) volume of at least 10 confocal z-stack images at 400 × magnification for each experiment performed using NIS elements software. MIF graphs represent VEGF time point fold change with respect to control vehicle treated NO-VEGF and the graphs bars represents the average of the a minimum of 3 separate experiments from HREC and HAEC. The results show that the nuclear translocating pool of the VEGFR2 in HREC are derived from the Golgi. * = *p* < 0.05 as determined by 2-way ANOVA followed by multi-comparison post-hoc Tukey’s test shows intra-group differences with respect to the VEGF treatment and # = *p* < 0.05 2-way ANOVA followed by multi-comparison post-hoc Tukey’s and t-student tests shows inter-group differences with respect to the Vehicle and Brefeldin A treatment.

Brefeldin A treatment did not influence the VEGF-stimulated translocation of VEGFR1 to the HREC nucleus (Fig. 7A and C), but it did decrease the nuclear translocation of VEGFR1 to HAEC nucleus after 10–30 min of VEGF treatment (Fig. 7A and C). In contrast to VEGFR1, the blockade of the biosynthetic protein pool from the ER to the Golgi completely abrogated the nuclear translocation of VEGFR2 in HREC (Fig. 7B–C), substantially decreasing the levels of this receptor in the nucleus at all time points after stimulation with VEGF. However, Brefeldin A did not influence the nuclear levels of VEGFR2 seen in VEGF-treated HAEC compared to vehicle control (Fig. 7B–C).

## Discussion

While it is well recognized that a wide range of RTKs, including VEGFRs, translocate to the nucleus where they regulate cellular functions^9,19,22,26–28^, the spatial and temporal intracellular translocation of VEGFR1 and VEGFR2 following ligand binding has not been fully elucidated. In this study we report that following VEGFA stimulation: (1) a rapid and substantial nuclear translocation of VEGFRs occurs; (2) VEGFR1 and VEGFR2 exhibit different spatial and temporal translocation characteristics both in vitro and in vivo which determine the nuclear VEGFR1:VEGFR2 ratio; (3) VEGFR translocation differs between microvascular and macrovascular cells; (4) VEGFRs in the nucleus are partially uncleaved and minimally phosphorylated; (5) VEGFR translocation is associated with the endosomal pathway and comes from Golgi; and (6) there is an increase in the volume of intracellular organelles.

We and others have demonstrated the nuclear localization of VEGFR1 and VEGFR2 in a variety of microvascular endothelial cells as well as neoplastic cells suggesting a strong association of nuclear VEGFR with pathological angiogenesis^4–6,8,9,16,17,29,30^. There have been limited studies^8,9,30^ assessing the temporal changes in nuclear VEGFRs following VEGF stimulation with time points ranging from 100 seconds to 48 h following VEGF treatment^8,9,16,30^ which is in keeping with this study and other RTKs such as EGFR^26^. We realize the ligand activation is a rapid process occurring in a matter of minutes^31^ but selected our time points based on: (a) our prior studies; (b) nuclear translocation would take longer than canonical receptor activation with a 17Kd protein estimated to take at least 2 min to pass from cytosol to nucleus^32^ but VEGFRs are likely to be longer still given that VEGFRs are around 180–200 KDa; (c) VEGFRs would be retained in the nucleus for a substantial period as we clearly show. Furthermore, our in vitro observations with microvascular endothelial cells are supported by our in vivo studies which showed nuclear translocation of VEGFRs following VEGF stimulation. Interestingly, nuclear localization of VEGFR2 was significantly different between micro- and macrovascular cells with microvascular cells showing strong nuclear translocation of VEGFR2 following VEGF stimulation while there was no significant nuclear translocation of VEGFR2 in macrovascular cells under the same conditions. Furthermore, while the VEGFR1:VEGFR2 ratio decreased in microvascular endothelial cells it increased in macrovascular endothelial cells after VEGF treatment. These differences are perhaps not surprising since, it is widely accepted that there are significant differences between different vascular beds and large versus small vessels^1,33^. Furthermore, VEGF can differentially induces VEGFR2/STAT3 complex formation and STAT3 activation in microvascular versus macro-vascular endothelial cells and that this correlates with VEGF stimulating its own gene expression via an autocrine mechanism^34,35^. Activation of VEGFR2 is known to be associated with cell proliferation, tumor progression and pathological angiogenesis^1,12,30,36,37^, however, studies show that its regulation and response can be dependent on VEGFR1, that can act as a negative regulator of VEGFR2^1,2^. Zhang and co-workers^30^ showed an inversely reciprocal pattern of VEGFR1 and VEGFR2 regulation in tumor-associated and endothelial cells where VEGF-induced angiogenesis is regulated by an interaction between both receptors. Previously, we reported^25^ that the VEGFR1:VEGFR2 ratio is important in maintaining the integrity of adherens junctions and coordinates vascular permeability. It should be noted that there was some donor-to-donor variation in the timing and extent of nuclear VEGFR translocation in the primary HREC cultures but in all cases VEGF stimulated nuclear translocation of VEGFRs. Such donor variation between primary cultures is well documented^38–40^.

Interestingly, whilst there are no significant temporal differences in nuclear translocation of the C- and N-terminal portions of VEGFR1 there was an 80% increase in the C-terminal staining for VEGFR1 compared to N-terminal staining. This would suggest that the nuclear translocation of VEGFR1 is primarily in a cleaved form. VEGFR1 cleavage has been reported by us and others^16,41^ and Notch signaling is the classic example of nuclear translocation of a cleaved receptor to regulate nuclear function^42^. The same is not true for VEGFR2 which showed a similar increase both C- and N-terminal portions in microvascular endothelial suggesting that, unlike VEGFR1, VEGFR2 migrates full length to the nucleus. However, further proteomic and sequencing studies would be required to ascertain the exact make up of these intranuclear VEGFRs. Irrespective of whether it is a C-terminal VEGFR fragment or a full length VEGFR, nuclear VEGFR1 and VEGFR2 are minimally phosphorylated indicating that the translocation of these receptors to the nucleus is independent of intracellular signaling/phosphorylation and their action in the nucleus is independent of phosphorylation.

To confirm that nuclear translocation was not a feature solely of cell culture we assessed if intravitreal injection of VEGF promoted nuclear translocation of VEGFRs in retinal microvascular endothelial cells. We clearly demonstrate that following VEGF there is significant translocation of both VEGFR1 and 2 to the nucleus although the translocation of nuclear VEGFR1 appeared to occur much faster than VEGFR2. This led to an increased VEGFR1:VEGFR2 ratio as we observed in our cell cultures. Although confirmatory, the in vivo study is limited by the restricted choice of anti-VEGFR antibodies available for immunoelectron microscopy and a single intravitreal injection of VEGF is insufficient to promote a neovascular response in the retina although it does promote retinal vascular permeability^25^.

Little is known about VEGFR1 internalization and intracellular traffic, but VEGFR1 is likely internalized through clathrin-mediated endocytosis via early endosome-GTPase and Rab4^43^. However, more is known about the internalization of VEGFR2^43–45^. Recently Basagiannis and co-workers^46^ described that the internalization of this receptor is via a clathrin-dependent pathway only in the absence of ligand while VEGF binding induces the internalization of VEGFR2 via micropinocytosis and this endocytic route is crucial for downstream VEGFR2 signaling and function in endothelial cells.

Our data regarding the intracellular and organelle distribution of VEGFR1/2 following VEGF activation indicates that: (1) C-terminal VEGFR1 nuclear translocation was not significantly impacted by blockade of receptor synthesis even though reduced localization of VEGFR1 was observed in the Golgi complex suggesting that VEGFR1 translocates to the nucleus from trans Golgi apparatus and early endosomes, probably leaving via the recycling pathway; (2) VEGFR2 nuclear translocation was minimal when the biosynthetic pool was inhibited and together with the strong temporal and spatial association with early endosomes indicates that VEGFR2 derives from Golgi after rapid biosynthesis and translocates to the nucleus via the early endosome-mediated pathway; and (3) post-signaling reduction of nuclear VEGFRs suggests that they can exit the nucleus and probably reenter the endosomal pathway for degradation as we see an increase co-localization of VEGFRs with lysosomes. It is well recognized that nuclear proteins are exported from the nucleus and can enter the proteosomal and lysosomal pathways and that there is significant interplay between these two pathways^47,48^. Molecular studies are needed to determine the mechanism(s) by which VEGFRs enter and exit the nucleus since they do not contain a nuclear localization/export signal. Studies on other RTKs such as EGFR and ErbB4 suggest that they “piggy-back” onto a protein with a nuclear translocation sequence or sumoylation may be important^26,49,50^. Furthermore, VEGFR2 has been shown to bind once in the nucleus VEGFR2 can regulate both binding of transcription factors such as Sp1 and VEGFR2 regulate its own transcription^9^. Thus the nuclear VEGFR1:VEGFR2 ratio may play a key role in regulating angiogenic outcome. Transfection of cells with VEGFRs containing a nuclear targeting sequence in the absence of VEGF may offer the best approach to determine the impact of nuclear VEGFR ratio on neovascular responses.

An unexpected observation was that VEGF stimulation increased organelle size. To our knowledge, this has not been previously reported but regulation of organelle size is important in modulating cellular activity^51,52^. However, the actual mechanism(s) regulating cell size remain an area of extensive research but given that cellular organelles are dynamic structures that respond to cellular changes their capacity is likely to be upregulated by cellular changes^53^. A classic example is the Golgi for which size is regulated by both intracellular and extracellular cues, often affects distinct functional zones and is associated with biosynthetic needs^53,54^. There is, to our knowledge, no evidence that VEGF, or other growth factors, directly regulate organelle size so the effect we observed is likely to be indirect and represent change in organelle needs in respect to VEGF-induced biosynthesis, endosomal transport requirements and degradation.

In conclusion, we have further elucidated the intracrine VEGF signaling pathway and shown that the nuclear translocation of VEGFRs is both receptor and vessels (macro versus micro) dependent. Our data indicates that the endosomal pathway plays a key role in the translocation of VEGFRs to the nucleus from the Golgi and the subsequent export to the lysosomal system. The significance of these findings is that the targeted trafficking of VEGFRs to, and maintenance of the ratio of VEGFR1:VEGFR2 at, intracellular compartments represents an important alternative VEGF signaling pathway. Based on our novel observations together with reports that VEGFRs bind transcription factors and regulate gene activity^9,34,35^ we hypothesize that VEGF-driven vascular permeability and neovascularization are highly dependent on the targeted subcellular translocation of specific VEGFRs and that the ratio of VEGFR1:VEGFR2 within subcellular compartments. This VEGFR pathway requires further investigation at the molecular level and may explain, in part, why extracellular anti VEGF therapy has limited success in a significant number of patients.

## Supplementary Information

Supplementary Information.
