# Supplementary material for: Spatial and temporal VEGF receptor intracellular trafficking in microvascular and macrovascular endothelial cells
Source: Sci Rep. 2021 Aug 30;11:17400. doi: 10.1038/s41598-021-96964-7 (PMC8405636; doi:10.1038/s41598-021-96964-7)
Supplement: Supplementary file 1 — Supplementary Information. [file 41598_2021_96964_MOESM1_ESM.pdf]

Supplementary Figure 1

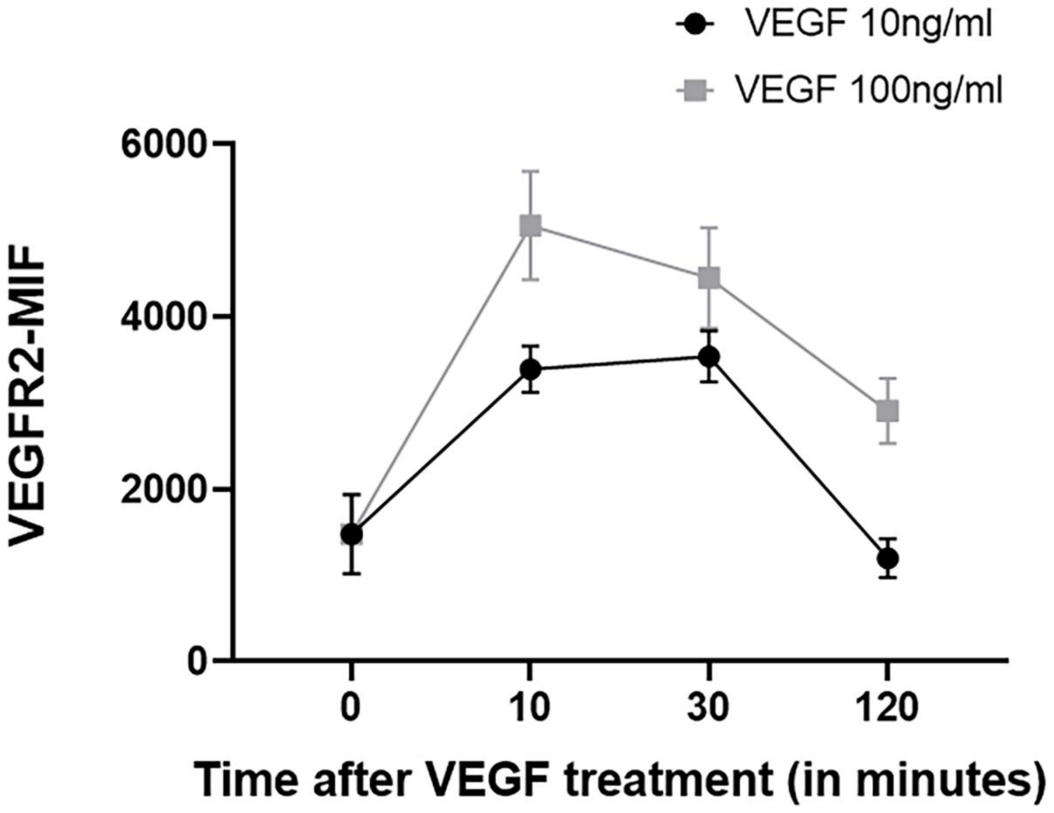

Supplementary Figure 1. Dose-dependent VEGFR nuclear translocation in endothelial cells. Graphical representation of nuclear migration of VEGFR2 after treatment with low (10ng/ml) and high (100ng/ml) VEGF doses. Graph show quantification of the median intensity of fluorescence (MIF) of VEGFR2 at least 10 confocal z-stack images at 400x magnification performed using NIS elements software. Data for each VEGF time point was normalized by the control NO-VEGF and the graph show the average ± SEM.

Supplementary Figure 2

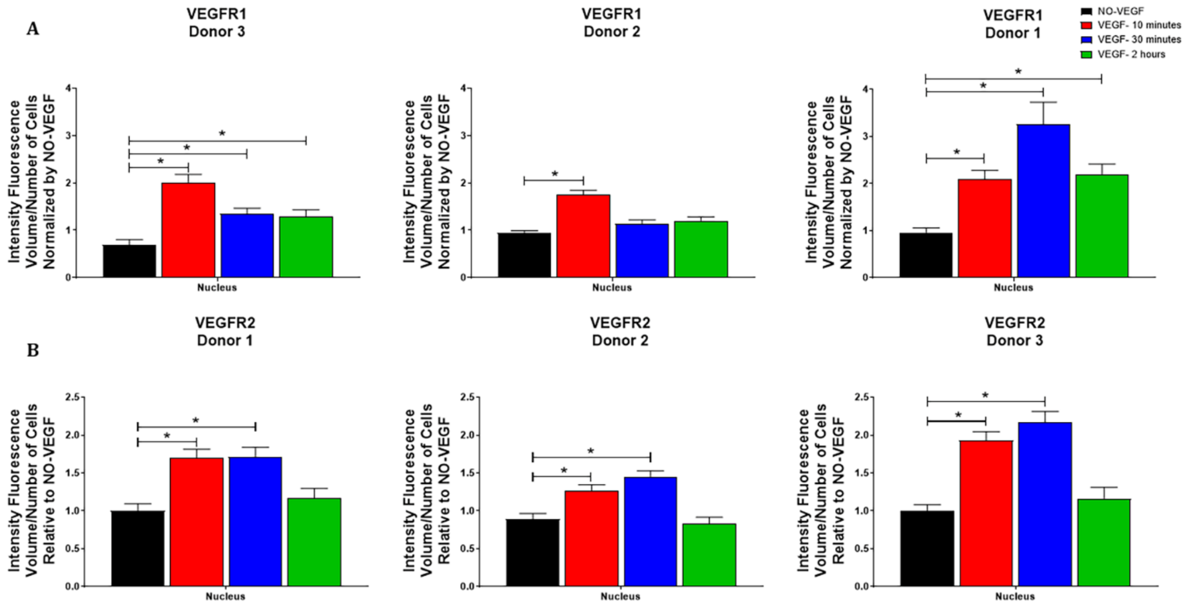

Supplementary Figure 2. Time-dependent VEGFR nuclear translocation profile between HRECs from 3 different donors. Graphs show quantification of the median intensity of fluorescence (MIF) volume of (**A**) VEGFR1 and (**B**) VEGFR2 at least 10 confocal z-stack images at 400x magnification for each experiment performed using NIS elements software. Data for each VEGF time point was normalized by the control NO-VEGF and the graphs show the average from a minimum of 3 separate experiments for each donor. For all graphs, bars show the average of the 3 independent experiments after normalization of the VEGFR1 or VEGFR2 to control without VEGF for each donor. * = $p<0.05$ as determined by 1-way ANOVA and multi-comparison post-hoc Tukey’s test is considered significant.
